# A Case of Pyogenic Vertebral Osteomyelitis and Iliopsoas Abscess Caused by Invasive Pneumococcal Disease Serotype 35F: Utility of Diffusion-Weighted Whole-Body Imaging With Background Body Signal Suppression as an Adjunctive Diagnostic Tool

**DOI:** 10.7759/cureus.87770

**Published:** 2025-07-12

**Authors:** Koichi Kimura, Koji Hayashi, Kosuke Misaki, Mamiko Sato, Yuka Nakaya, Toshiko Iwasaki, Yasutaka Kobayashi

**Affiliations:** 1 Department of Rehabilitation Medicine, Fukui General Hospital, Fukui, JPN; 2 Department of Orthopedics, Fukui General Hospital, Fukui, JPN; 3 Department of Radiology, Fukui General Hospital, Fukui, JPN; 4 Graduate School of Health Science, Fukui Health Science University, Fukui, JPN

**Keywords:** diffusion-weighted imaging (dwi), dwibs, iliopsoas abscess, invasive pneumococcal disease, mri images, pyogenic vertebral osteomyelitis, streptococcus pneumoniae

## Abstract

We present a case of invasive pneumococcal disease (IPD) complicated by both iliopsoas abscess and pyogenic vertebral osteomyelitis caused by serotype 35F *Streptococcus pneumoniae* in a 64-year-old man with a history of splenectomy who was unvaccinated. The patient experienced difficulty moving, severe back pain, vomiting, and high fever. Advanced imaging techniques, including T2-weighted lumbar MRI and diffusion-weighted whole-body imaging with background body signal suppression (DWIBS), revealed hyperintensities in the left iliopsoas muscle and L4-L5 vertebral bodies, facilitating diagnosis. Blood cultures confirmed the presence of serotype 35F *S. pneumoniae*, a non-vaccine type associated with an increased risk of invasive disease and mortality. The patient was successfully treated with targeted antibiotics and disc lavage, resulting in symptom resolution. To our knowledge, this is the first reported case of serotype 35F *S. pneumoniae *causing both iliopsoas abscess and vertebral osteomyelitis, making it a noteworthy contribution to medical literature. Notably, DWIBS proved to be a valuable adjunct diagnostic tool, highlighting its potential for visually accessible, comprehensive screening of inflammation and abscesses throughout the body. We believe that DWIBS is particularly useful when bacteria capable of inducing lesions in multiple organs, such as *S. pneumoniae* or* Staphylococcus aureus, *are isolated from blood cultures. Although DWIBS is still emerging in infectious disease diagnostics, this case underscores its promising role in detecting abscesses and inflammatory lesions.

## Introduction

*Streptococcus pneumoniae* (*S. pneumoniae*) is a major global pathogen, particularly affecting children and the elderly, and is a leading cause of death in children under five [1]. Globally, it causes approximately one million deaths annually, mainly from pneumonia in Africa and Asia [2,3]. *S. pneumoniae* is a primary cause of otitis media, complicated pneumonia, meningitis, and septicemia/septic shock. Invasive pneumococcal disease (IPD) encompasses severe infections like bacteremia, sepsis, meningitis, and osteomyelitis, where *S. pneumoniae *is isolated from sterile sites [4]. Thus, S*. pneumoniae* can cause infections and abscesses throughout the body, which may occur simultaneously in some cases.

*S. pneumoniae* is one of the capsule-forming bacteria. The bacterium's polysaccharide capsule, which protects it from host defenses, determines its serotype [5]. Over 100 serotypes exist among* S. pneumoniae* [6]. Pneumococcal serotype influences clinical and epidemiological factors, with certain serotypes (e.g., 1, 2, 4, 5, 7F, 8, 9, 12F, 14, 16, 18C, 19A) being more invasive, while others (e.g., 3, 6A, 6B, 11A, 15B/C, 19, 23F) are less so [7,8]. Serotypes also vary in association with specific syndromes, with serotypes 1 and 3 more commonly isolated in pneumonia, and serotypes 6, 10, and 23 in meningitis [7,8]. Globally, serotypes 14 and 19A are frequent IPD isolates [7,9].

Diffusion-weighted whole-body imaging with background body signal suppression (DWIBS) is an advanced functional magnetic resonance imaging (MRI) technique, based on diffusion-weighted imaging (DWI), that enables the evaluation of tissues and their microenvironment by assessing the Brownian motion of water molecules, without the need for contrast media [10,11]. It provides a strong contrast between areas with restricted diffusion (e.g., inflammation, tumors) and normal surrounding tissues [11,12]. The technique employs a short tau inversion recovery (STIR) echo-planar imaging (EPI) sequence with free breathing to acquire multiple thin-slice DWI images [10-13]. Background signals from normal tissues are suppressed using fat suppression and heavy diffusion weighting, improving the detection of abnormalities [10-12]. Images can be presented as 3D PET-like images [10], and DWIBS images can be fused with T2-weighted images for enhanced anatomical assessment [12]. DWIBS offers advantages over PET-CT or contrast-enhanced CT due to its lack of radiation exposure, absence of contrast agent requirement, and cost-effectiveness [10,11,13].

In this report, we describe a case of IPD with pneumococcal vertebral osteomyelitis (PVO) and iliopsoas abscess (IPA) caused by serotype 35F pneumococci, in which DWIBS was used as a supportive diagnostic tool alongside conventional modalities such as blood and tissue cultures, computed tomography (CT), and MRI.

## Case presentation

**Figure 1:**
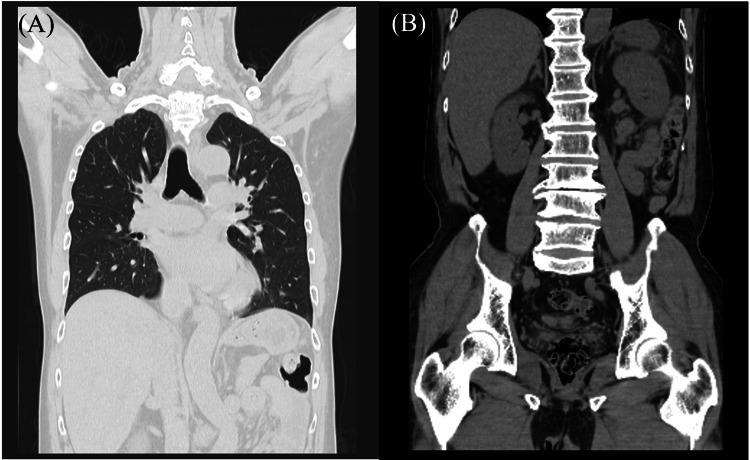
The results of computed tomography (CT) without contrast agent. A: chest CT revealed no obvious abnormal shadows in the lung fields; B: abdominal CT showed no abnormality in vertebrae or iliopsoas muscles.

**Figure 2:**
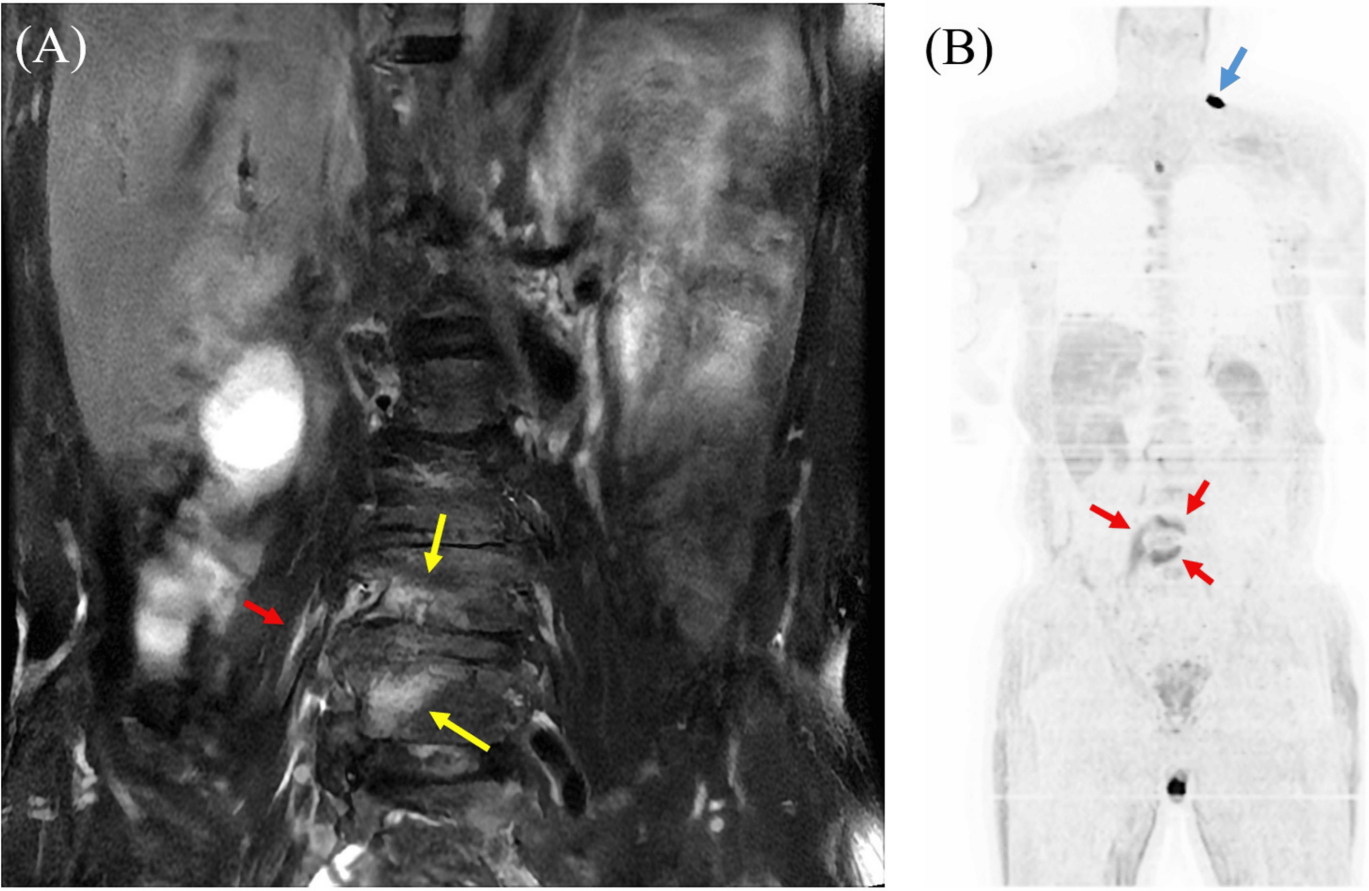
The result of magnetic resonance imaging (MRI) using diffusion-weighted whole-body imaging with background body signal suppression (DWIBS) and T2-weighted MRI with fat suppression. A: T2-weighted MRI with fat suppression revealed hyperintensity in the L4 and L5 vertebral bodies (yellow arrows) and iliopsoas muscle (red arrow), consistent with the DWIBS hyperintensity lesions (indicated by arrows); B: MRI on DWIBS showing hyperintensities in the L4 and L5 vertebrae and right iliopsoas muscle (red arrows) as well as skin surface around left neck (blue arrow). The advantage of DWIBS is that it can visually screen for abscesses and tumors throughout the body.

A 64-year-old male developed difficulty in movement and severe back pain upon waking. His symptoms persisted in the afternoon, and he developed vomiting and a fever of 40°C, leading him to visit our emergency department. He had a history of splenectomy at age seven and hypertension in his 50s; however, he had no history of pneumococcal vaccination. Upon admission, his vital signs indicated clear consciousness, a blood pressure of 125/74 millimeters of mercury, a pulse rate of 83 beats per minute, and a body temperature of 40.3°C. Neither nuchal rigidity nor jolt accentuation was observed. Muscle strength and superficial sensation were preserved. Blood tests revealed elevated leukocyte count, C-reactive protein (CRP) level, blood sugar, hemoglobin A1c, urea nitrogen, aspartate aminotransferase, lactate dehydrogenase, and creatine phosphokinase, as well as decreased hemoglobin, total protein, albumin, sodium, potassium, and chloride (Table 1). A chest-abdominal computed tomography (CT) scan without contrast was unremarkable, including in the vertebral body and iliopsoas muscle (Figures 1A, 1B). Lumbar MRI showed hyperintensities in the L4 and L5 vertebral bodies on T2-weighted imaging (Figure 2A). DWIBS demonstrated hyperintensities in the L4-5 vertebrae and the right psoas muscle (Figure 2B). Transthoracic echocardiography revealed mild mitral regurgitation, aortic regurgitation, and tricuspid regurgitation, but no abnormalities such as vegetations were observed.

**Table 1:** The results of blood tests on admission. IFCC: International Federation of Clinical Chemistry and Laboratory Medicine

Inspection Items	Result	Reference Range
Red blood cells (RBC)	401×10⁴/μL	(435-555×10⁴)
White blood cells (WBC)	11600/μL	(3300-8600)
Hemoglobin	12.3 g/dL	(13.7-16.8)
Platelets	13.4×10⁴/μL	(15.8-34.8×10⁴)
Blood glucose	138 mg/dL	(73-109)
Hemoglobin A1c	6.2%	(4.6-6.0)
Total protein	6.4 g/dL	(6.6-8.1)
Albumin	2.9 g/dL	(4.1-5.1)
Blood urea nitrogen (BUN)	23.1 mg/dL	(8-20)
Creatinine	1.11 mg/dL	(0.65-1.07)
Total bilirubin	1.3 mg/dL	(0.4-1.5)
Aspartate aminotransferase (AST)	88 U/L	(13-30)
Alanine aminotransferase (ALT)	20 U/L	(10-42)
Alkaline phosphatase (ALP) (IFCC method)	68 U/L	(38-113)
Lactate dehydrogenase (LDH)	335 U/L	(124-222)
Gamma-glutamyltransferase (γ-GTP)	15 U/L	(13-64)
Cholinesterase (ChE)	219 U/L	(201-421)
Creatine phosphokinase (CPK)	6464 U/L	(59-248)
Sodium (Na)	133 mmol/L	(138-145)
Potassium (K)	3.5 mmol/L	(3.6-4.8)
Chloride (Cl)	99 mmol/L	(101-108)
Calcium (Ca)	8.0 mg/dL	(8.8-10.1)
Triglycerides	45 mg/dL	(40-234)
Total cholesterol	117 mg/dL	(142-248)
High-density lipoprotein cholesterol (HDL cholesterol)	68 mg/dL	(38-90)
Low-density lipoprotein cholesterol (LDL cholesterol)	40 mg/dL	(65-163)
C-reactive protein	19.66 mg/dL	(0.00-0.14)

He was treated with ceftriaxone 2 g per day. On day five, two sets of blood cultures grew *S. pneumoniae* (serotype 35F; the serotype of *S. pneumoniae *was identified at the Department of Bacteriology 1, National Institute of Infectious Diseases, Tokyo, Japan). He was diagnosed with IPD complicated by IPA and PVO. Because the isolated *S. pneumoniae* was sensitive to penicillin G (PCG), treatment with 16 million units of PCG per day was initiated. On day six, an L4/5 disc lavage was performed, and purulent fluid was obtained; however, the Gram stain and culture were negative. He was treated with PCG for four weeks, followed by amoxicillin (AMPC) for three weeks, which led to the resolution of his symptoms, including fever and back pain.

## Discussion

To our knowledge, this case represents the first reported instance of serotype 35F *S. pneumoniae* associated with IPA and vertebral osteomyelitis. The patient had a history of splenectomy and non-vaccination, including the pneumococcal conjugate and polysaccharide vaccines. It is well-known that splenectomy increases susceptibility to encapsulated organisms, including *S. pneumoniae*, *Haemophilus influenzae*, and *Neisseria meningitidis* [14]. However, because serotype 35F is a non-vaccine type (NVT) [15], it remains unclear whether this IPD could have been prevented even if the patient had received the above-mentioned vaccine. The diagnosis of IPA and pyogenic vertebral osteomyelitis was not evident on CT scan but was clearly demonstrated by MRI, particularly using the DWIBS sequence. The isolated serotype 35F *S. pneumoniae *was highly sensitive to penicillin; therefore, the patient was treated with PCG followed by AMPC, and his clinical course was favorable. 

Serotype 35F of* S. pneumoniae*, classified as NVT, has gained increasing recognition, especially in southern Sweden, where it accounted for about 5% of clinical respiratory isolate samples after PCV13 introduction [15]. It is a prevalent NVT in mucosal infections, frequently found alongside other NVTs such as 11A, 23B, 15A, and related serotypes 15B and 15C, suggesting potential serotype replacement [15]. The serotype exhibits high nonsusceptibility to multiple antibiotics, notably penicillin, and often displays multidrug-resistant and extensively drug-resistant phenotypes, complicating treatment strategies [15].

Additionally, serotype 35F is associated with increased IPD risk, particularly among individuals with comorbidities or immunosuppression, with odds ratios of 3-5 and over 10, respectively, in Swedish data from 2006-2015 [16]. Post-vaccine surveillance indicates a rising incidence among vulnerable populations, reflecting serotype replacement [17]. Mortality studies from Denmark and Scandinavia reveal that infections caused by 35F are linked to higher short-term mortality, especially in bacteremia cases, with approximately 13.7% 30-day mortality in some cohorts, and a trend towards increased fatality following PCV13 rollout [16,17]. Overall, serotype 35F presents significant clinical challenges due to its antimicrobial resistance, increased disease severity, and impact on vulnerable groups.

PVO is considered a rare disease [18,19]. Historically, pneumococci were identified as the causative organism in only about 1.3% of vertebral osteomyelitis cases [20]. However, more recent studies suggest that its incidence might be underestimated, with one study identifying PVO in 6.4% of adult patients with IPD [19]. It is particularly noted in community-onset cases without a history of invasive procedures or back injury [19]. Previous studies reported that patients with PVO are often of older age, with a median age of 69 [19,21]. Common comorbidities include diabetes mellitus, heavy alcohol intake, and systemic lupus erythematosus (SLE), especially with prolonged corticosteroid use, which can lead to immune defects; a history of spinal surgery (e.g., lumbar disc herniation); and recent respiratory tract infections (e.g., tonsillitis, bronchitis, otitis media, mastoiditis, sinusitis, or pneumonia) [18-20,22]. These can act as the portal of entry for *S. pneumoniae*, which then spreads hematogenously [20,22].

Pneumococcal IPA is a rare but significant complication that often co-occurs with vertebral osteomyelitis [19,20,22-24], with patients frequently presenting with severe low back pain [18]. A positive psoas sign may be observed, indicating inflammation extending to the psoas muscle [22]. The pathophysiology of IPA involves either direct invasion from adjacent vertebral osteomyelitis or hematogenous spread from a distant infectious source [22]. Diagnostic imaging modalities such as MRI and CT scans are valuable tools for identifying IPA, revealing characteristic features of abscess formation [18,22,23]. The definitive diagnosis is confirmed through microbiological cultures demonstrating *S. pneumoniae* from blood samples or abscess drainage fluids [18,19]. Management typically involves a combination of targeted antibiotic therapy and abscess drainage, which can often be performed under CT guidance [18,20,22]. To the best of our knowledge, this represents the first reported case of serotype 35F *S. pneumoniae* causing both PVO and IPA, making it a particularly noteworthy contribution to medical literature.

In this case, DWIBS was used as a supportive diagnostic tool because it enables comprehensive detection of lesions throughout the body, particularly abscesses. While DWIBS can detect abscesses, tumors, and inflammation [10-13,25], its application has been primarily focused on tumor staging, with limited research exploring its utility in detecting abscesses and inflammation. To the best of our knowledge, regarding infectious diseases, there are only three reports of acute cholecystitis, acute focal bacterial nephritis, and myocardial abscess [11,12,26]. We believe that DWIBS may have potential applications in infectious diseases, particularly in detecting bacteria capable of producing metastatic lesions, such as *Staphylococcus aureus* or *S. pneumoniae*. Additionally, DWIBS may also be useful in the diagnosis of fever of unknown origin by aiding in the identification of the fever source. 

## Conclusions

To the best of our knowledge, we report the first case of serotype 35F *S. pneumoniae* causing both IPA and PVO in a splenectomized, unvaccinated patient. This case highlights the increasing clinical significance of serotype 35F, an NVT, which is associated with increased IPD risk, antimicrobial resistance, and higher mortality, especially in vulnerable populations. Furthermore, our case demonstrates the utility of DWIBS for diagnosing deep-seated infections like IPA and PVO, suggesting its potential broader application in infectious disease diagnostics and emphasizing the need for further research.
